# Assessing the Validity and Reliability of the PNO$\bar{E}$ for Measuring Cardiometabolic Outcomes During Walking Exercise

**DOI:** 10.3390/jfmk10020159

**Published:** 2025-05-06

**Authors:** Manny M. Y. Kwok, Shamay S. M. Ng, Jonathan Myers, Billy C. L. So

**Affiliations:** 1 Department of Rehabilitation Sciences, The Hong Kong Polytechnic University, Hong Kong, China; manny.kwok@connect.polyu.hk (M.M.Y.K.); shamay.ng@polyu.edu.hk (S.S.M.N.); 2 VA Palo Alto Health Care System, Stanford University, Palo Alto, CA 94304, USA; drj993@aol.com

**Keywords:** portable metabolic system, accuracy assessment, aerobic capacity, kinesiology

## Abstract

**Background:** The accuracy of measurement of cardiometabolic outcomes in terms of gaseous exchange and energy expenditure of individuals is crucial. The objective of this study was to compare the validity and reliability of the PNO$\bar{E}$ in measuring cardiometabolic outcomes from the respiratory gaseous exchange of healthy individuals during treadmill walking exercise. **Methods:** A total of 21 healthy subjects (15 male and 6 female) aged 22.76 ± 3.85 years took part in this study. Oxygen uptake (VO_2_), carbon dioxide production (VCO_2_), respiratory exchange ratio (RER), metabolic equivalents (METs), tidal volume (VT), and energy expenditure (EE) were measured using the PNO$\bar{E}$ and COSMED K5 portable systems during a twenty-eight-minute, four-stage incremental protocol, where speed increased from 1.7 mph to 4.2 mph with a 2% incline on a treadmill. Test–retest reliability was tested on separate days with trail repetition. Validity was evaluated by Bland–Altman plots, intraclass correlation coefficients (ICCs) and mean percentage difference. **Results:** ICCs showed that VCO_2_ was in the good range (0.75–0.90). The ICC of the RER from stages 1 to 3 of the incremental protocol and the VT from stages 2 to 4 of the incremental protocol showed good to excellent reliability. No clear trend was seen for VO_2_, VCO_2_, and EE datapoints with variations in speed. Pearson’s correlation coefficients were moderately high (r = 0.60–0.79) between VO_2_, VCO_2_, RER, METs, VT, and EE measured by the PNO$\bar{E}$ and K5 systems. All subjects, except for a few cases in VT, were within the upper and lower 95% confidence intervals of the acceptable range of the Bland–Altman plots. **Conclusions:** The PNO$\bar{E}$ system is a valid and reliable measure of cardiometabolic outcomes and is comparable to the COSMED K5 system.

## 1. Introduction

Cardiometabolic outcomes are commonly evaluated using metabolic systems [1]. Metabolic analysis, commonly termed cardiopulmonary exercise testing (CPET), is a powerful test for the early detection of predisposing factors for major chronic cardiometabolic diseases. According to the American Heart Association, CPET provides a strong predictor of longevity and has been the gold standard for personalizing nutrition, training, and recovery programs for many years [2]. Parameters such as oxygen consumption (VO_2_), carbon dioxide production (VCO_2_), energy expenditure (EE), tidal volume (VT), respiratory exchange ratio (RER), and metabolic equivalents (METs) are commonly used to assess cardiometabolic function and determine fitness levels, and have numerous clinical applications. CPET responses are readily available to therapists and sports scientists for monitoring long-term cardiometabolic fitness and designing exercise regimes for health and performance purposes [3].

Cardiometabolic data can be measured using either metabolic carts or portable devices. Accurate measurement is essential to reflect the true cardiometabolic function of individuals across the health and disease spectrum. Technological advancements have facilitated the transition from laboratory-based measurements to portable systems, enabling measurement of metabolic data in real time outside of the laboratory [4]. This shift is important as laboratory testing can be cumbersome, labor-intensive, and expensive [5]. Overcoming these limitations is crucial to promoting widespread application of CPET assessments and necessitates the development of portable systems that are valid, reliable, practical, and cost-effective for evaluating various sports performances.

A comprehensive review conducted by Macfarlane et al. [1] emphasized that the validity and reliability of current high-technology portable metabolic systems are not widely established, and only a few independent studies have been published [1]. While portable metabolic carts like the COSMED K5 allow for the measurement of the same variables as stationary metabolic carts, there is evidence suggesting lower accuracy [6]. Therefore, there is a need for the development of small and portable, yet valid and reliable, metabolic analyzers to quantify cardiometabolic variables [7]. 

One such newly developed portable metabolic device is the PNO$\bar{E}$ system. It is designed to measure cardiometabolic outcomes under both laboratory and field conditions. The PNO$\bar{E}$ device features an electrochemical fuel cell sensor for oxygen measurement, a non-dispersive infrared sensor for carbon dioxide measurement, and a proprietary hot film anemometer sensor for flow measurement. The PNO$\bar{E}$ device operates in a breath-by-breath mode, allowing for continuous measurement of volume and simultaneous determination of expired gas concentrations. The advantage of this technology is its stable construction and absence of moving parts, making it suitable for use in the PNO$\bar{E}$ system.

There has been one independent study conducted to evaluate the validity and reliability of the PNO$\bar{E}$ system compared to a previously validated stationary metabolic cart (COSMED QUARK-CPET), which reported that the portable system can accurately determine respiratory gases over a range of exercise intensities [8]. However, to the best of our knowledge, there has not been a study evaluating cardiometabolic responses to exercise (VO_2_, VCO_2_, VT, MET, EE, RER) of the PNO$\bar{E}$ system compared to a portable metabolic cart. To address this knowledge gap, the objectives of this study were to address the validity and reliability of the PNO$\bar{E}$ (ENDO Medical Inc., Palo Alto, CA, USA, https://pnoe.com/ (accessed on 2 March 2025)) in measuring cardiometabolic responses (VO_2_, VCO_2_, RER, VT, MET, EE) among healthy individuals during a treadmill walking protocol. It is hypothesized that the accuracy and reliability of the PNO$\bar{E}$ is comparable to a portable metabolic cart in measuring cardiometabolic data during exercise.

## 2. Materials and Methods

### 2.1. Participants

Twenty-one young healthy adults (fifteen men, six women) were recruited through posters around university campus to participate in this mixed-design study. The inclusion criteria were adults who self-reported as healthy, free of cardiorespiratory disease, not taking any medications, and aged between 20 and 35 years. The exclusion criteria included subjects who had self-reported cardiorespiratory or neurological pathology and/or had an orthopedic fracture or any surgical intervention performed on the lower extremities in the six months prior to the study. The proposed time frame could probably capture relevant outcomes while allowing enough time for healing and recovery, if any. All participants were informed of the study risks by an investigator, ethical approval was obtained, and they signed an informed consent form prior to data collection.

### 2.2. Study Design and Procedures

Participants underwent an initial screening interview and familiarization process before providing informed consent for the study. The International Physical Activity Questionnaire was administered to assess participants’ physical activity levels for descriptive purposes [9].

All participants completed a familiarization session one week prior to the study, during which they practiced using the mouthpiece and a Hans Rudolph valve to reduce measurement errors. A standardized mouthpiece was used for all participants, and they received feedback and instructions during the trial. Participants were required to attend two sessions at the university laboratory with a gap of 2–7 days between sessions. The laboratory temperature and humidity were recorded during each visit. During the first visit, resting heart rate (HR), blood pressure (BP), body mass (kg), and height (cm) measurements were taken. Subsequently, participants completed a 28 min walking protocol on a treadmill while wearing the portable cardiometabolic device. Proper fitting of the metabolic equipment was confirmed by closing the valve of the mask and checking for air leakage through maximal expiration before each test. The fit was continuously monitored during breath-by-breath measurements to ensure there was no lifting of the mask indication of leakage.

The protocol began with 5 min of walking at 1.7 mph with a 10% inclination, followed by a 2 min rest period. Participants stepped on the treadmill during the walking phase and rested by stepping off the treadmill during the rest phase. This 5 min walking and 2 min rest cycle was repeated as the walking speed increased to 4.2 mph with a 16% inclination [10]. Breath-by-breath data were collected continuously throughout the procedure. Participants performed the walking protocol twice, wearing the two proposed devices in a randomized and counterbalanced order.

### 2.3. Instruments

The PNO$\bar{E}$ device operates on lithium batteries and weighs approximately 800 g. The device is composed of a single housing (120 × 110 × 45 mm, height, width, length, respectively), fastened to a shoulder harness and carried by the participant throughout exercise. Participants wore a standard Hans Rudolph mask with mouthpiece and breathed through a flow sensor (Hans Rudolph Inc., Kansas City, MO, USA). The flow sensor, positioned in the breathing circuit, measures the volume and flow rate of exhaled air while simultaneously analyzing the concentrations of O_2_ and CO_2_ using infrared spectroscopy or electrochemical sensors. The flow meter uses ultrasonic transducers positioned across the airflow path to send and receive high-frequency sound waves. The transit time of ultrasonic pulses is measured in both directions (with and against the airflow) [8]. The difference in transit time is proportional to the flow velocity, allowing precise calculation of the instantaneous flow rate (L/min).

The COSMED K5 (firmware version 1.1) is a portable metabolic cart designed to be worn using an anatomical harness. The device features a Galvanic fuel cell O_2_ sensor and digital infrared CO_2_ sensor. The system transmits data via Bluetooth telemetry to the analysis software for visualization in real time. Features that facilitate the field use of the K5 include GPS and an altimeter. It allows measures of gas exchange responses including VO_2_, VCO_2_, RER, VT, MET, and EE.

### 2.4. Validity Assessment

The validation protocol was conducted using an incremental approach based on a modified Bruce protocol [11]. Each stage lasted for five minutes, starting from 1.7 mph with a 10% incline, then progressing to 2.5 mph with a 12% incline, followed by 3.4 mph with a 14% incline, and finally reaching 4.2 mph with a 16% incline. The walking intensity was monitored by visually inspecting the heart rate response and the Borg Scale score. The intensity levels were classified to reflect relatively light to moderate cardiometabolic demands, following the guidelines of the American College of Sports Medicine [12]. Cardiometabolic variables were recorded using values from the final minute of each five-minute stage. Measurements were obtained simultaneously using both the COSMED-K5 system and the PNO$\bar{E}$ (ENDO Medical, Palo Alto, CA, USA) alternatively, with the COSMED-K5 serving as the reference standard to ensure accuracy and provide consistent, comparable data.

### 2.5. Reliability Assessment

The reliability of the PNO$\bar{E}$ device was evaluated by measuring cardiometabolic variables on the selected subjects with the same walking protocol, on separate days. The same experimental setup was followed in both visits. The test was performed at the same time of day and subjects were instructed to wear similar clothing and the same walking shoes for the two trials.

### 2.6. Statistical Analysis

The normality assumption was stated, and the Shapiro–Wilk test was also conducted to assess it. All results are presented as mean values and standard deviation (mean ± SD). Statistical significance was accepted at the 5% level (*p* ≤ 0.05). Reliability was assessed by calculating the coefficient of variation (CV). The test–retest reliability of the PNO$\bar{E}$ system was assessed through a two-way random-effects (consistency) analysis of variance (ANOVA) model and reported as intraclass correlation coefficients to determine agreement between the two metabolic carts. A 95% confidence interval (CI) was used to describe the variability in the ICC. An ICC of 0.50–0.75 indicated moderate reliability, good reliability was identified as an ICC = 0.75–0.90, and excellent reliability was considered to be an ICC > 0.90 [13]. Validity analysis of the PNO$\bar{E}$ system was assessed by comparing the data collected by the PNO$\bar{E}$ and COSMED K5. Student’s paired sample t tests were used to compare differences between the variables measured by PNO$\bar{E}$ and COSMED K5. To identify bias, an absolute mean difference was calculated. Pearson’s correlation coefficients (r) were used to evaluate the relationship between the devices. A moderate relationship was identified as r = 0.40–050, moderately high as r = 0.60–0.79, and high as r ≥ 0.80 [14]. Bland–Altman plots of the differences between PNO$\bar{E}$ and COSMED K5 were plotted against the average of the two measures [15]. All analyses were performed using the Statistical Package for the Social Sciences (SPSS), Version 23.0, for Windows.

## 3. Results

Fifteen men and six women were recruited, and all successfully completed the reliability and validity tests. The descriptive statistics for the study sample are presented in Table 1. The mean age of the participants was 22.76 ± 3.85 yrs. Their average height was 169.38 ± 9.69 cm, average weight was 64.19 ± 11.92 kg, and average BMI was 22.20 ± 2.58. The average physical activity levels derived from the IPAQ were 28.6% at level 1, 19.0% at level 2, and 52.4% at level 3.

### 3.1. Reliability

Overall, the coefficient of variation (CV) was low for HR, VT, and RER at the faster walking speeds and high at the slower walking speeds despite RER showing a high CV at the fastest walking speed. However, no specific patterns were seen on VO_2_, VCO_2_, and EE for the changes in the CV with speed variations. The CV ranged from 3.4% to 20.8% for all variables across all speeds (Table 2). The ICC was found to be moderate (0.50–0.75) across HR levels 1–2, level 4 of RER, and level 1 of VT. For the rest of the variables at all speeds, the ICC generally remained at a good level 0.75–0.9. In particular, the ICC found in levels 3 and 4 of the VT was higher than 0.9, indicating excellent reliability. Most of the ICCs for VCO_2_ were in the good range (0.75–0.90). The ICCs for RER from levels 1 to 3 and VT for levels 2–4 were in the good-to-excellent reliability range.

### 3.2. Validity

**Figure 1:**
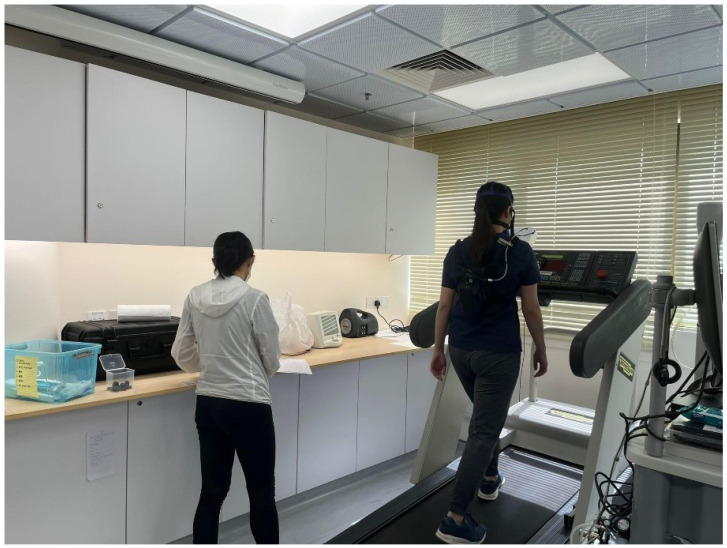
Experimental setup.

**Figure 2:**
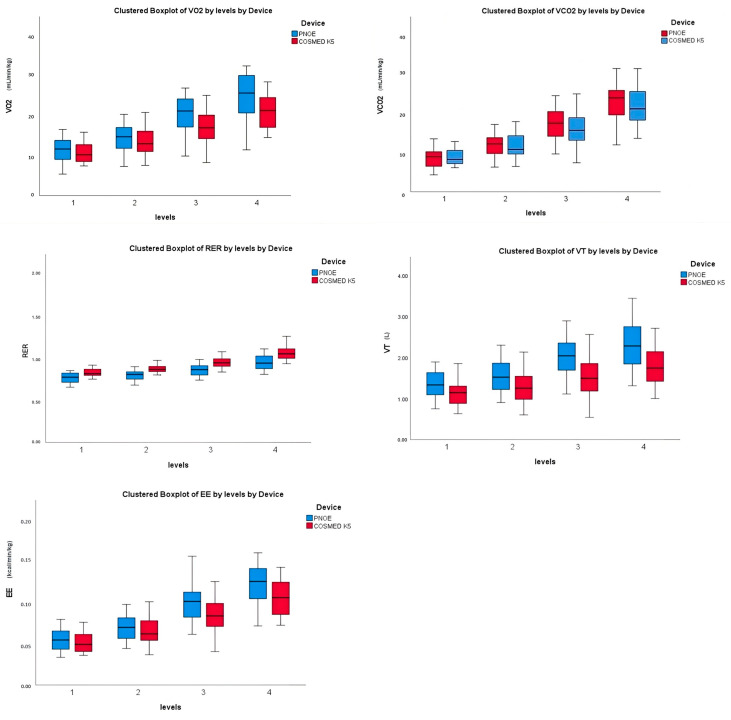
Comparison of cardiometabolic variables across levels of exercise spectrum between PNO$\bar{E}$ and COSMED K5. Box and whisker plot present median, 25th and 75th percentiles for metabolic variables including VO_2_, VCO_2_, RER, VT, and EE.

There were no significant differences between the mean values for HR, VO_2_, VCO_2_, RER, VT level 3, and EE levels 1–3 when comparing the PNO$\bar{E}$ and K5 device. However, there were significant differences between the three walking speeds in VT and one walking speed in EE (Figure 1). The mean VT was significantly higher (*p* < 0.05) at speeds in levels 1, 2, and 4 when measured by the PNO$\bar{E}$ compared with the K5. Similarly, the mean EE was significantly higher (*p* < 0.05) at the highest speed of level 4 when comparing the PNO$\bar{E}$ with the K5 (Figure 2).

Correlation coefficients were moderately high (r = 0.60–0.79) between metabolic variables measured by the PNO$\bar{E}$ and K5 systems, with the exception of RER, which had values outside the moderate range. Correlation coefficients were in the high range (r ≥ 0.80) between metabolic variables in level 3–4 HR and level 4 VT measured by the PNO$\bar{E}$ and K5 systems (Table 3).

The absolute mean percentage difference revealed that PNO$\bar{E}$ tended to have a bias toward higher values for all metabolic variables compared with the K5 system. Scores for the absolute mean percentage difference ranged between 0.01 and 25.5%. The greatest absolute mean percentage difference was observed at all speeds for the VT, particularly the fastest speed (4.2 mph, Level 4). RER exhibited the lowest mean absolute mean percentage differences (ranging from 0.01 to 0.05) of all the metabolic variables.

**Figure 3:**
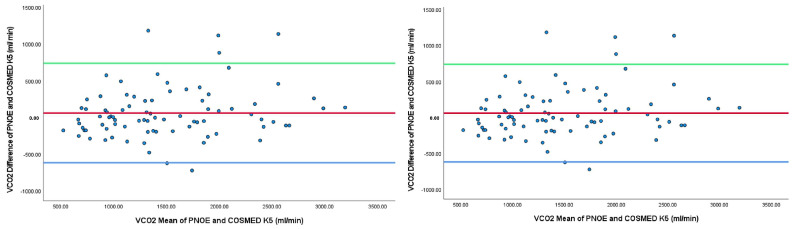

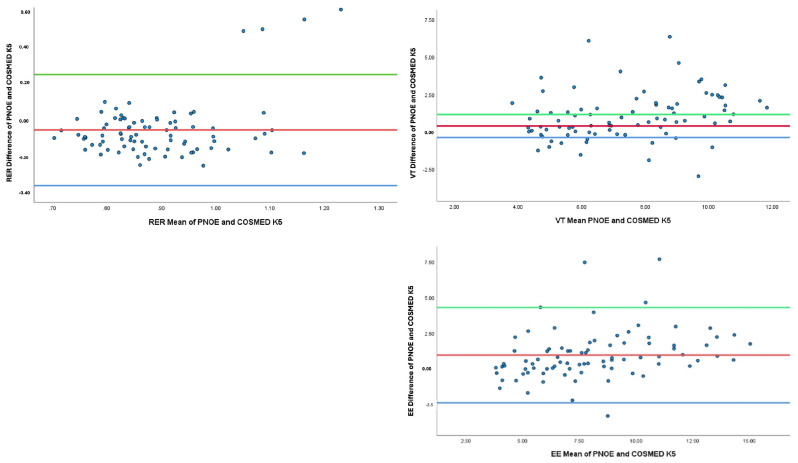
Agreement between PNO$\bar{E}$ and COSMED K5 for VO_2_, VCO_2_, RER, VT, and EE. **Red line:** Mean reference line for agreement; Green and Blue line: Limit reference line of agreement.

Bland–Altman plots for the relationships between the two systems are shown in Figure 3. The Bland–Altman plots look for evidence of proportional bias. It was a simple way to evaluate a bias between the mean differences, and to estimate an agreement interval, within 95% of the differences in the two metabolic carts of PNO$\bar{E}$ and COSMED K5. Cases outside the upper and lower confidence intervals should also be noted.

## 4. Discussion

In this study, we address the validity and reliability of the PNO$\bar{E}$ (ENDO Medical, Palo Alto, CA, USA) in measuring cardiometabolic responses (VO_2_, VCO_2_, RER, VT, MET, EE) among healthy individuals during a treadmill walking protocol. The reliability of the metabolic variables was assessed using the CV. Overall, the CV values for all metabolic variables were relatively small, indicating good reliability. Lower CV values, particularly for RER and VT at higher speeds, were considered more favorable as they indicated less variability around the mean [16]. VT was measured via a graded exercise test, during which VO_2_ and VCO_2_ were continuously monitored. The PNO$\bar{E}$ system assessed VT in real time using breath-by-breath analysis, with data processed through proprietary algorithms and displayed on the PNO$\bar{E}$ software interface [17]. While the VT showed moderate reliability at slower speeds, the PNO$\bar{E}$ device demonstrated good to excellent reliability in measuring VO_2_, VCO_2_, RER, METs, and EE at higher speeds. These findings suggest that the PNO$\bar{E}$ device may be less reliable at slower speeds compared to other speeds, possibly due to greater variations in step length and increased metabolic costs associated with slower speeds [18]. Consequently, this could potentially result in lower reliability for the PNO$\bar{E}$ device at slower speeds.

Ensuring reliable and valid measurements is crucial when using portable metabolic devices to assess cardiometabolic outcomes during exercise. To the best of the authors’ knowledge, this is the first study aimed at investigating the validity and reliability of the PNO$\bar{E}$ device compared to the COSMED K5 across different treadmill walking speeds in relation to cardiometabolic data. Previous research has evaluated the reliability and validity of the PNO$\bar{E}$ device against the COSMED Quark CPET, a stationary metabolic cart [19]. The COSMED Quark CPET or an automated online system operate within a reliable measurement range of 4–12% [20]. The findings of that study indicated that the PNO$\bar{E}$ portable metabolic cart is comparable in accuracy to the stationary metabolic cart, capable of precisely measuring respiratory variables across a wide range of exercise intensities under laboratory conditions in healthy adults [8]. Differences in expired gas volume measured by the K5 and PNO$\bar{E}$ systems may arise from several factors, including variations in sensor calibration and the different algorithms used for calculation. Additionally, individual participant variability in breathing patterns and tidal volume may affect the volume of expired gas captured [21].

Similarly, evidence supports the accuracy of the COSMED K5 when compared to a metabolic cart during submaximal cycling exercise. The COSMED K5 demonstrated comparable accuracy to the metabolic cart in measuring VO_2_, VCO_2_, RER, and EE, although it slightly underestimated VO_2_ and VCO_2_ by 6–7% [22]. This discrepancy could be attributed to several factors, such as differences in sensor calibration, measurement techniques, or algorithms used by each device. When comparing the COSMED K5 to the K4b2, a moderately strong relationship was observed in VO_2_, VCO_2_, and EE across a range of walking speeds. Additionally, a strong correlation was found for VO_2_ and VCO_2_ between the PNO$\bar{E}$ device and the COSMED-CPET metabolic cart, with the PNO$\bar{E}$ device demonstrating satisfactory repeatability [19]. However, it is important to note that RER measurements may yield slightly different results due to variations in measurement and algorithms. Differences in measurement methodologies, such as variations in the accuracy and calibration of gas analyzers and the time response of sensors at low-to-moderate exercise intensities, can contribute to variations in RER measurement [23]. Therefore, it is important to consider the specific characteristics and limitations of determining RER.

In terms of validity, we utilized a Bland–Altman plot analysis to evaluate agreement between the PNO$\bar{E}$ and COSMED K5 portable metabolic analyzers. Our findings revealed good agreement with an acceptable bias between the two devices for VO_2_, VCO_2_, RER, METs, and EE across the four levels of the incremental protocol. The Bland–Altman plots demonstrated that the differences between the two devices remained consistent across the entire range of exercise intensities, indicating similarity. However, for VT, there was a lack of similarity as the values fell outside the upper and lower 95% confidence intervals between the two devices. It is important to note that VT is an absolute measure that tends to minimize individual variability, resulting in a restricted spread of values for this variable. Previous research by Leprete et al. (2012) also reported a strong relationship between respiratory variables obtained simultaneously using two commercially available portable metabolic systems [24]. They found no significant differences in measures of VO_2_ and VT; however, there was a notable difference in RER and VCO_2_ at maximal exercise intensity. These differences could be attributed to participants’ characteristics or mask properties, such as dead space and resistance to airflow. Another potential explanation for these discrepancies could be methodological differences between the protocols, such as treadmill versus cycling.

The validity findings strongly indicate that the PNO$\bar{E}$ metabolic system is acceptable for measuring VO_2_, VCO_2_, RER, MET, and EE across the four levels of incremental exercise intensities. Although there were significant differences in VT measurements between the two devices, the magnitude of the difference was minimal. This discrepancy may be attributed to the PNO$\bar{E}$ device’s sensitivity to ambient CO_2_ levels, highlighting the impact of environmental factors on the PNO$\bar{E}$‘s ability to reliably and accurately assess metabolic data [25]. These results provide strong support for the utilization of the PNO$\bar{E}$ portable metabolic system for measuring cardiometabolic data throughout various stages of incremental exercise intensities. The observed r values for VO_2_, VCO_2_, VT, and EE indicated moderately high associations (r = 0.60–0.79). These findings are somewhat consistent with previous studies comparing VO_2_ measurements between the PNO$\bar{E}$ and a metabolic cart [19]. The significant correlations observed between VO_2_, VCO_2_, VT, and EE provide further support for the validity of measuring cardiometabolic responses using both the PNO$\bar{E}$ and COSMED K5 devices.

These findings align with previous research that highlights the significance of aerobic contributions to the energy demands of an incremental walking protocol [25]. Assessing the aerobic contribution in a graded walking protocol is valuable as it helps avoid potential issues related to mask displacement and thermal strain. This insight can provide exercise specialists and coaches with important considerations regarding the role of aerobic measurements using different portable metabolic devices. The moderate-to-high associations observed between the PNO$\bar{E}$ and COSMED devices is consistent with the experimental design of PNO$\bar{E}$ and COSMED-Quark CPET, which are designed to examine cardiometabolic responses to exercise. It is worth noting that in the current study, a strong relationship (r = 0.86) was observed between metabolic variables, particularly VT, at higher speeds when measured by both the PNO$\bar{E}$ and K5 systems. The strong relationship of VT at higher speeds measured by the PNO$\bar{E}$ and K5 systems may stem from consistent measurement techniques, increased respiratory demand, compatible algorithms, similar participant characteristics, and a controlled testing environment.

This study is novel in several respects. The evaluation of aerobic power and metabolic outcomes is important for exercise specialists as it provides a fundamental baseline measurement and serves as a monitoring and motivational tool to evaluate exercise progression. Understanding that the reliability and validity results of both the PNO$\bar{E}$ and COSMED K5 systems were comparable to each other can inform objective measurement considerations for these portable metabolic devices. This practical evaluation process will greatly assist sports scientists in providing individualized exercise prescriptions. Therefore, the development of a reliable and accurate test for assessing metabolic outcomes using the PNO$\bar{E}$ and COSMED K5 portable metabolic devices is an essential step in evaluating cardiometabolic fitness of individuals seeking to increase their aerobic power for sports performance or overall health improvement.

This study has several limitations. First, food intake was not strictly controlled or measured prior to the assessments, which may have affected participant compliance and potentially influenced the measured metabolic variables. Additionally, not all participants performed the assessments at the same time of day, which could have introduced variability in the outcomes between participants. Furthermore, it is important to acknowledge that the participants in this study were healthy and physically active adults. Therefore, generalizing the true validity of the PNO$\bar{E}$ and COSMED K5 systems using these data should be approached cautiously, as temporal changes related to physical training or overall health status may have influenced the results. Another limitation is that the study only investigated treadmill walking, and it is important to explore other modes and intensities of exercise. Future research should involve testing the accuracy of the systems using different modes of exercise at various intensities under different environmental conditions.

## 5. Conclusions

The PNO$\bar{E}$, a portable metabolic device, demonstrated good-to-excellent test–retest reliability. It exhibited comparable performance to the COSMED K5 in measuring cardiometabolic variables across different exercise intensities. Notably, the PNO$\bar{E}$ showed moderate reliability specifically for RER at higher speeds. Overall, the PNO$\bar{E}$ was found to be valid when compared to the COSMED K5 for all variables except for RER.

## Figures and Tables

**Table 1:** Descriptive characteristics of participants (mean ± SD).

	Total (*n* = 21)	Women (*n* = 6)	Men (*n* = 15)
Age (years)	22.76 ± 3.85	21 ± 3	23.47 ± 3.93
Height (cm)	169.38 ± 9.69	157.67 ± 3.77	174.07 ± 6.99
Weight (kg)	64.19 ± 11.92	50.17 ± 2.61	69.8 ± 9.28
BMI (kg/m^2^)	22.20 ± 2.58	21.86 ± 1.44	22.33 ± 2.90
IPAQ level 1 (Low)	28.6% (6/21)	0% (0/6)	40% (6/15)
IPAQ level 2 (Moderate)	19.0% (4/21)	33.3% (2/6)	13.3% (2/15)
IPAQ level 3 (High)	52.4% (11/25)	66.7% (4/6)	46.7% (7/15)

IPAQ: International Physical Activity Questionnaire; Level 1: low physical activity; Level 2: moderate physical activity; Level 3: high physical activity; BMI: body mass index.

**Table 2:** Test–retest reliability of metabolic variables with PNO$\bar{E}$ (*n* = 21).

Bruce Protocol	Level 1 1.7 mph × 10%	Level 2 2.5 × 12%	Level 3 3.4 × 14%	Level 4 4.2 × 16%
HR (bpm)				
Visit 1 Visit 2 ICC (95% CI) CV (%)	111.47 ± 14.70 113.06 ± 12.16 0.51 (0.12–0.77) * 12.4	123.28 ± 17.46 123.43 ± 12.50 0.65 (0.32–0.84) * 10.5	141.44 ± 19.48 142.35 ± 15.38 0.84 (0.64–0.93) * 7.4	163.97 ± 21.20 165.21 ± 16.61 0.90 (0.77–0.96) * 5.5
VO_2_ (mL/min/kg)				
Visit 1 Visit 2 ICC CV (%)	18.03 ± 5.59 17.65 ± 5.91 0.80 (0.57–0.91) * 18.9	24.41 ± 6.96 22.66 ± 6.97 0.82 (0.61–0.92) * 15.9	31.52 ± 8.81 30.84 ± 9.33 0.86 (0.68–0.94) * 16.2	39.27 ± 10.91 37.79 ± 11.68 0.81 (0.58–0.92) * 19.0
VCO_2_ (mL/min/kg)				
Visit 1 Visit 2 ICC CV (%)	13.66 ± 4.43 13.79 ± 4.90 0.81 (0.59–0.91) * 18.4	19.32 ± 5.71 18.28 ± 5.84 0.87 (0.70–0.94) * 15.1	26.77 ± 7.72 26.77 ± 8.26 0.86 (0.69–0.94) * 15.1	36.81 ± 10.39 36.15 ± 11.12 0.82 (0.61–0.92) * 17.9
RER				
Visit 1 Visit 2 ICC CV (%)	0.76 ± 1.07 0.79 ± 0.13 0.79 (0.55–0.91) * 10.3	0.80 ± 1.03 0.82 ± 0.13 0.85 (0.67–0.94) * 8.0	0.85 ± 1.09 0.88 ± 0.14 0.85 (0.66–0.94) * 7.8	0.97 ± 0.18 0.97 ± 0.15 0.51 (0.11–0.77) * 17.5
Tidal Volume (L)				
Visit 1 Visit 2 ICC CV (%)	1.24 ± 0.38 1.35 ± 0.34 0.72 (0.43–0.88) * 20.8	1.56 ± 0.34 1.56 ± 0.41 0.88 (0.73–0.95) * 11.4	1.98 ± 0.52 2.01 ± 0.53 0.93 (0.84–0.97) * 8.1	2.35 ± 0.60 2.35 ± 0.60 0.99 (0.98–1.0) * 3.4
EE (kcal/min/kg)				
Visit 1 Visit 2 ICC CV (%)	0.08 ± 0.03 0.08 ± 0.03 0.81 (0.59–0.92) * 15.8	0.12 ± 0.03 0.11 ± 0.03 0.83 (0.62–0.93) * 13.9	0.15 ± 0.04 0.14 ± 0.04 0.88 (0.72–0.95) * 12.5	0.19 ± 0.05 0.18 ± 0.05 0.81(0.58–0.92) * 16.0

ICC: Intraclass Correlation Coefficient; 95%CI: 95% Confidence Interval; * *p* < 0.05.

**Table 3:** Validity of the PNO$\bar{E}$ for select metabolic variables compared with K5 (*n* = 21).

Bruce Protocol	Level 1 1.7 mph × 10%	Level 2 2.5 mph × 12%	Level 3 3.4 mph × 14%	Level 4 4.2 mph × 16%
HR (bpm)				
Pearson’s r				
Absolute Mean % Difference	0.57 * 0.55	0.74 * 1.50	0.89 * 0.37	0.95 * 0.18
VO_2_ (mL/min/kg)				
Pearson’s r				
Absolute Mean % Difference	0.67 * 6.54	0.65 * 6.07	0.53 * 12.90	0.58 * 14.00
VCO_2_ (mL/min/kg)				
Pearson’s r				
Absolute Mean % Difference	0.66 * 1.40	0.67 * 0.66	0.55 * 5.50	0.62 * 5.49
RER				
Pearson’s r				
Absolute Mean % Difference	0.12 0.01	−0.07 0.03	−0.30 0.04	−0.27 0.05
VT (L)				
Pearson’s r				
Absolute Mean % Difference	0.62 * 17.16	0.74 * 16.67	0.62 * 21.39	0.86 * 25.53
EE (kcal/ min/kg)				
Pearson’s r				
Absolute Mean % Difference	0.69 * 6.97	0.69 * 5.97	0.58 * 13.07	0.63 * 14.40

* *p* < 0.05.

## Data Availability

Data will be made available upon reasonable request to the corresponding author.
